# New 2-D silver(I) coordination network constructed from thiomethyl group-substituted *p-tert*-butylthiacalix[4]arene

**DOI:** 10.55730/1300-0527.3459

**Published:** 2022-05-11

**Authors:** Huriye AKDAŞ-KILIÇ, Ernest GRAF, Mir Wais HOSSEINI, Nathalie KYRITSAKAS

**Affiliations:** 1 Department of Chemistry, Yıldız Technical University, İstanbul, Turkey; 2 Universite de Rennes, CNRS, ISCR-UMR, ISCR-UMR, Rennes, France; 3 Laboratoire de Tectonique Moléculaire, UMR CNRS, icFRC, Strasbourg University, Strasbourg

**Keywords:** Molecular networks, coordination polymer, thiacalix[4]arene, X-ray structure, thioether

## Abstract

New ligand based on *p*-*tert*butyltetrathiacalix[4]arene blocked in 1,3-alternate conformation, was achieved via a multistep synthesis with the introduction of thiomethylpropoxy groups on the lower rim which leads to a neutral tecton. The combination of this *p*-*tert*butyltetrathiacalix[4]arene **(*p*-TCA-2**), in 1, 3-alternate imposed conformation, with tetrahedral Ag(I)SbF_6_ salt, leads to the formation of neutral, new 2-D coordination network, which was structurally investigated in the solid state by X-ray diffraction methods on a single crystal.

## 1. Introduction

In the field of chemistry, nanotechnology results in the construction of nanometer-sized materials by synthetic means or the interconnection of chemical molecules. Thus, to obtain functional materials, it is necessary to be able to synthesize molecules capable of transferring information through their electronic, optical, or ionic properties. Finally, it is necessary to be able to assemble them in a perfectly controlled way in order to obtain a material having not only the intrinsic properties of the molecules that compose it but also the properties of the material. Obtaining materials with specific properties requires the arrangement of these molecules within the material, in order to take full advantage of their individual properties (alignment of dipole moments, magnetic moments, etc.). The design of molecular architecture in the solid-state is actually a challenging issue of current interest. Molecular tectonics, the research area dealing with the design and generation of such architectures [1] is a powerful strategical approach, which relies on concepts such as molecular recognition, developed in supramolecular chemistry [2] for which the components are assembled by attractive intermolecular interactions. Indeed, molecular tectonics concerns the control, under self-assembly conditions, of connectivity between informed and programmed molecular building blocks or tectons through iterative molecular recognition processes [3–8]. A further step, which remains to be achieved, consists of the incorporation of physical properties within the molecular tectons in order to obtain a functional network. Indeed, the variety of specific features such as coordination geometry, redox, optical, and magnetic properties associated with metallic centers by using extensively coordination bonds and their incorporation within coordination polymers [9, 10] or metal organic frameworks [11] (metals as structural nodes of the network), may lead to new materials, obtained by self-assembly processes, and lead to possible applications [12].

The number of translations of the assembling cores into one, two, or three directions of space respectively will define the dimensionality of the network, i.e. 1-, 2- or 3- D [13–16]. The majority of coordination networks reported are based on the use of cationic metal centers. In order to get neutral networks in the case of neutral organic tectons, the presence, of either an anionic organic tecton or the presence of anions is required. For the latter case, the self-assembly processes taking advantage of the presence of the anion to direct the dimensionality were previously reported [17].

For the design of tectons, *p-tert*-butyltetrathiacalix[4]arene (*p*-TCA), which is a macrocyclic organic platform, composed of four phenol moieties connected by thioether bridges is of interest because of steric reasons, this macrocycle is not planar but adopts four limit conformations (cone, partial cone, *1,2-alternate* and *1,3-alternate*) [18]. Among the four limit conformations adopted by TCA, the *1,3-alternate* conformer is particularly interesting because of four coordinating sites, since the alternate fashion allows to positioning of four interactions below and above the macrocyclic backbone. The *p-tert*-butylthiacalix[4]arenes have been decorated both at the upper and/or lower rims with a variety of monodentate donor sites and periodic infinite 1-, 2- and 3-D silver coordination networks based on thiacalix[4]arene or *p-tert*-butylthiacalix[4]arene derivatives in 1,3-alternate conformation bearing four nitrile groups [19], carboxylate units [20] or benzonitrile groups [21] have been described. Combinations of thiacalix[4]arenes derivatives bearing carboxylate groups with metal cations and auxiliary ligands lead to infinite architectures [22]. To the best of our knowledge, 2-D network with *p-tert*-butylthiacalixarenes bearing pendant thiomethylpropoxy groups has never been reported previously. In the present contribution, we report a new 2-D coordination network from ***p-*TCA-2** combined with Ag(I)SbF_6_ salt and we demonstrate the influence of the flexibility of the coordinating arm on the dimensionality of the network by X-ray diffraction studies.

## 2. Material and methods

### 2.1. Experimental

^1^H and ^13^C NMR were obtained on a Bruker WP 200 SY (200 MHz) and Bruker VC 300 (300 MHz) at 298 K. The deuterated solvent was used as purchased. X-ray diffraction data collection was carried out on a Kappa CCD diffractometer equipped with an Oxford Cryosystem liquid N_2_ device, using graphite-monochromated Mo-Ka radiation. For all structures, diffraction data were corrected for absorption and analysed using the OpenMolen package [23]. All non-H atoms were refined anisotropically.

### 2.2. Synthesis

General. All reagents were purchased from commercial sources and used without further purification. *P-tert*butylbutylthiacalix[4]arene (*p*-TCA) was prepared by using previously reported procedures [24].

#### 5,11,17,23-Tetrakis(*tert-*butyl)- 25,26,27,28-tetra(3-bromopropyloxy) tetrathiacalix[4]arene

A suspension of *p*-*tert*butylthiacalix[4]arene (1 g, 1.3 mmol), acetone (60 ml) and K_2_CO_3_ (1.5 g, 0.1 mmol) is stirred at 80°C for 3 hours. After adding 10 ml of dibromopropane, the suspension is stirred at the same temperature for 3 days. After filtration, the solvent is evaporated off and the residue is washed with 30 ml of MeOH to give 1.3 g of pure product ***p*-TCA-1**.

Yield: 92%, Colorless solid. mp: 310 °C. C_52_H_68_O_4_Br_4_S_4._1/2C_5_H_12_ (requires: C, 52.93; H, 6.06; S, 10.28 found: C, 52.74; H, 6.01; S, 10.33 %). ^1^H NMR (CDCl3): δ =1.32 (s, 36H, C(C*H*_3_)_3_), 1.54 (q, *J* = 7.3 Hz, 8H, C*H*_2_), 3.07 (t, *J* = 7.1 Hz, 8H, C*H*_2_), 3.97 (t, *J* = 6.8 Hz, 8H, C*H*_2_), 7.35 (s, 8H, Ar*H*). ^13^C NMR (CDCl3): δ = 30.4, 31.4, 32.2, 34.4, 67.0, 127.2, 128.2, 146.5, 156.3.

#### 5,11,17,23-Tetrakis(*tert-*butyl)- 25,26,27,28-tetra(3-thiomethylpropyloxy) tetrathiacalix[4]arene

A suspension of (3-tetrabromopropyloxy)-*p*-*tert*butylthiacalix[4]arene ***p*-TCA-1** (0.5 g, 4.14 mmol), in the presence of NaSCH_3_ (200 mg, 2.8 mmol) in THF (20 ml) is refluxed overnight. The mixture was then brought to room temperature, the solvent evaporated and the residue triturated with MeOH to give 410 mg of product ***p*-TCA-2**.

Yield: (93% yield). Colorless solid. mp: 315 °C (decomp). C_56_H_80_O_4_S_8_

^1^H NMR (CDCl3): δ = 1.30 (s + m, 44H, C(C*H*_3_)_3_ + C*H*_2_), 2.04 (s, 12H, C*H*_3_), 2.26 (t, *J* = 7.2 Hz, 8H, C*H*_2_), 3.92 (t, *J* = 7.5 Hz, 8H, C*H*_2_), 7.33 (s, 8H, Ar*H*). ^13^C NMR (CDCl3): δ = 15.9, 28.5, 31.1, 31.3, 34.3, 67.7, 127.5, 128.0, 145.8, 156.7.

### 2.3. Crystallisation conditions

#### Procedure for ligand

In a crystallization tube (4 mm diameter, 15 cm height), a solution of compound ***p-*TCA-2** (5.5 mmol) in degassed CHCl_3_ (1 mL) was layered carefully with a degassed MeOH (1 mL). Slow diffusion at room temperature produced crystals suitable for X-ray diffraction studies after several days.

#### Procedure for Network

In a crystallisation tube (4 mm diameter, 15 cm height), a solution of compound ***p-*TCA-2** (5.5 mmol) in degassed dioxane (1 mL) was layered with a degassed dioxane/MeOH (1/1) mixture (1 mL). A solution of AgSbF_6_ (5.5 mmol) in degassed MeOH (1 mL) was carefully added. Slow diffusion at room temperature produced crystals suitable for X-ray diffraction studies after several days.

### 2.4. Single-crystal XRD data

Table. Crystallographic data for Networks **A** and **B**

## 3. Results and discussion

**Figure 1:**
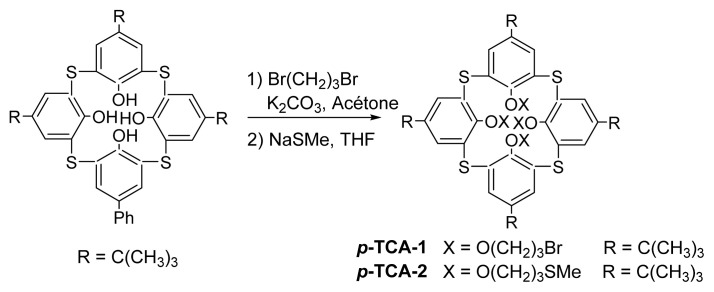
Synthesis of *p*-TCA-1 and *p*-TCA-2 derivatives.

Metallo-organic frameworks, widely described in the literature, are numerous and the complexes obtained can take the form of linear segments, helicoidal structures, rack structures, ladders, or grids formed by the assembly of rigid tectons or macrocycles via coordination bonds [25]. For the design of this tecton we use the same criteria as for previous works [26] based on *p*-*tert*butyltetrathiacalix[4]arene, namely a ligand presenting two-by-two divergent thioether type coordination sites on the lower rim in a blocked 1,3-alternate conformation. Compound ***p*-TCA-2** including all these *ab initio* design information, presented in Figure 1, has been prepared via a multi-step synthesis and characterized.

**Figure 2:**
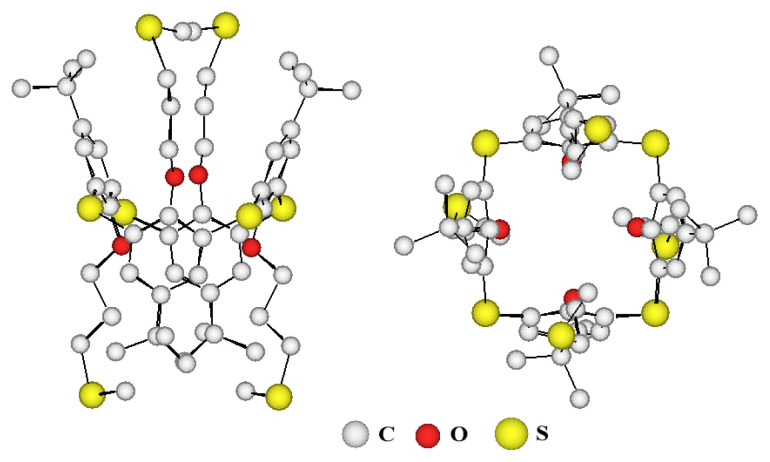
X-ray crystal structure of the ligand ***p*-TCA-2** Left: side view; Right: top view.

*P*-*tert*butyltetrathiacalix[4]arene was initially synthesized according to a known procedure [18]. The synthesis of this type of ligand was carried out in two steps (Figure 1), starting from *p-tert*butylthiacalix[4]arene ***p-*TCA** which was initially treated with an excess of dibromopropane in acetone under reflux, in the presence of K_2_CO_3_ to give the tetrabrominated intermediate ***p*-TCA-1** in very good yields and in 1,3-alternate conformation (Figure 1). These kinds of compounds carrying four bromopropyl chains can be qualified as precursors. Indeed, bromo groups are very good nucleofuges, they can be replaced by all types of nucleophilic groups and thus may give a very wide variety of thiacalix[4]arenes in 1,3-alternate conformation exclusively, ***p*-TCA-1** can be considered as an important intermediate. This intermediate, in the presence of sodium methanethiolate in refluxing THF, yields quantitatively tecton ***p*-TCA-2** (93 %) and was identified by NMR and X-ray diffraction (Figure 2). The solid-state X-ray characterization confirms the 1,3-alternate conformation for compound ***p*-TCA-2**.

The thiacalix[4]arene derivative ***p*-TCA-2** adopts the 1,3-alternate conformation positioning, thus two opposite units on the same face of the thiacalix platform, which was confirmed also by ^1^H NMR spectroscopy. Indeed, the protons in the thiomethyl group are shifted to 1,34 ppm compared to the tables (≈ 2 ppm). These groups, because of the 1,3- alternate conformation, are under the shielding effect of the aromatic wall. The macrocycle presents coordinating sites in two opposite directions which can allow the formation of a coordination network based on the use of the above-mentioned strategy. In the cases when the coordinating arms are rigid and linear, we will observe one-dimensional coordination network. [27]

This intermediate, in the presence of sodium methanethiolate in refluxing THF, yields quantitatively the tecton ***p*-TCA-2** which was characterized by X-ray diffraction on a single crystal (Figure 2) obtained by slow diffusion of a MeOH solution into a CHCl_3_ solution containing product ***p*-TCA-2**. The X-ray crystal structure shows that the thiacalix[4]arene derivative is blocked in 1,3-alternate conformation. The average distance observed for the C-S bond is 1.76 Å, with a CSC angle of 107.6°. We can also notice an average bond distance for C_Ar_-O of 1.36 Å and 1.63 Å for S-Me bonds.

Among several trials dealing with the formation of coordination networks using tecton *p*-**TCA-2**, we attempted the complexation of all these ligands with various metal salts, AgSbF_6_ salts, so far, have given single crystals of suitable quality for X-ray diffraction which was structurally characterized in the solid-state. At room temperature, upon slow diffusion of a dioxane solution of ***p-*TCA-2** into a MeOH solution of AgSbF_6_, colourless single crystals were obtained respectively after ca. 48 h and were studied by X-ray diffraction. Crystal information data are summarized in the Table.

**Figure 4:**
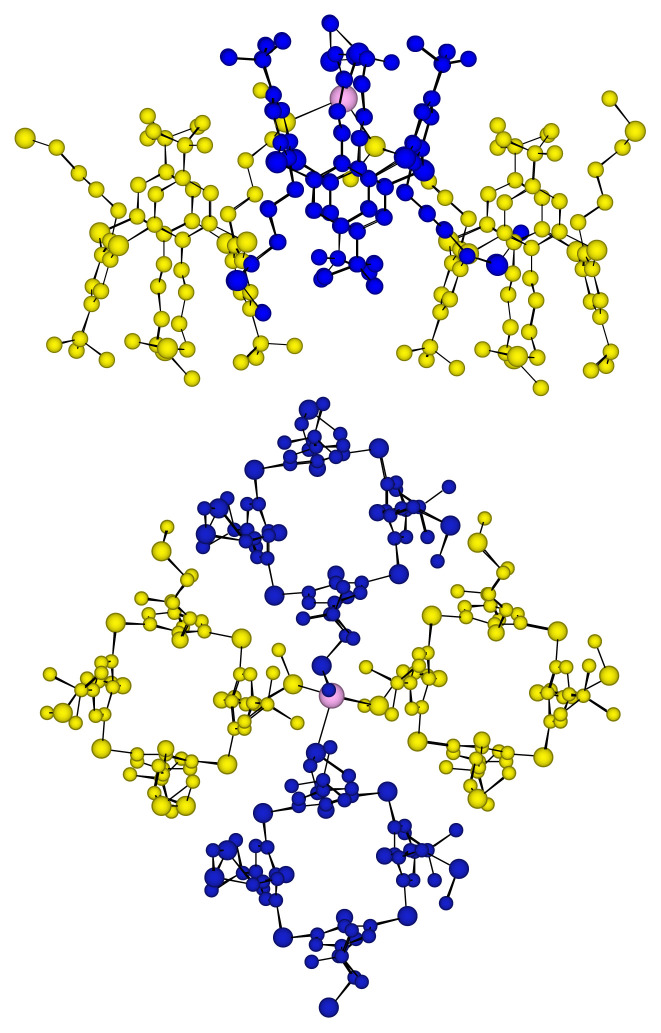
Disposition of the ligands ***p*-TCA-2** around the Ag center. Top: side view; Bottom: front view (SbF_6_^−^ anions omitted for clarity).

X-ray crystallographic analysis shows that the crystal is composed of a ***p-*TCA-2** ligand and Ag(I) atoms and the two-dimensional molecular network can be described by an infinite association of ligands and metals in two directions of the space. The crystal (monoclinic system, space group P 1 21/n 1) is composed of charged networks and SbF_6_^−^ anions. The metal Ag(I), which can adopt linear or tetrahedral coordination geometries, in this network adopts a tetrahedral coordination geometry, is bonded to the sulfur atoms of four different thiacalix[4]arenes and this part represents the elementary unit of the coordination network and one thiacalix[4]arene derivative ***p*-TCA-2** is bonded to four different silver atoms and his center is located at the top of a square of 13.8 Å side, which by translation makes it possible to build the 2-D network (Figure 4). The compensation of the positive charges of the Ag(I) cations is made possible by the presence of SbF_6_^−^ anions in the interstices of the crystal, the latter have been omitted for more clarity.

**Figure 5:**
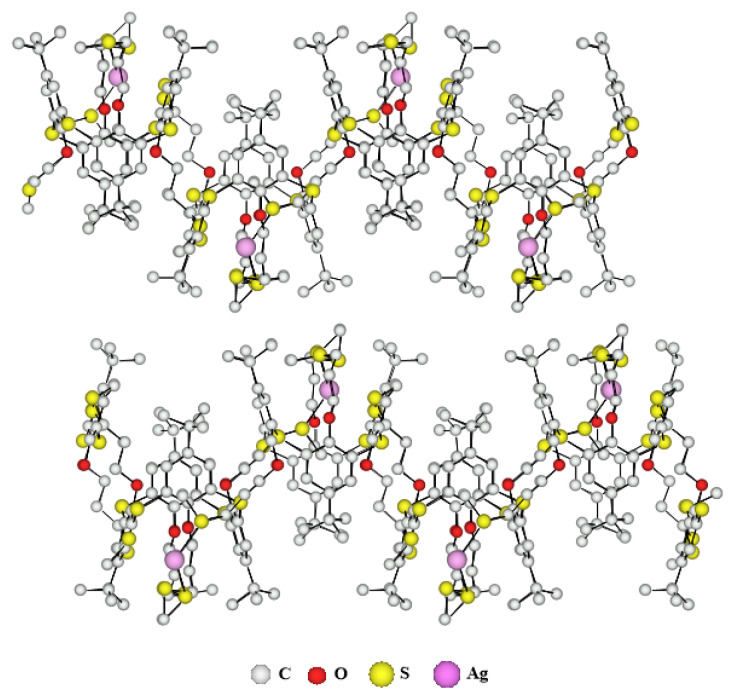
Part of the X-ray crystal structure of the 2-D network obtained with ligand ***p*-TCA-2** and AgSbF_6_, stacking of the layers in the crystal (SbF_6_^−^ anions omitted for clarity).

The tetrahedral coordination geometry of the Ag(I) cation is slightly distorted since the S-Ag-S angles vary from 117.2° to 127.2° and the CSC angles are on average 106.5° whereas it was 107.6 ° for the free ligand. We observe an average distance for the Ag-S bond is 2.61 Å and an average bond distance for C-S of 1.72 Å and 1.69 Å for S-Me bonds. The four thiacalix[4]arenes derivatives ***p*-TCA-2** around an Ag(I) cation can be grouped in pairs. Indeed, a front view of the elementary unit (Figure 5) shows that two thiacalix[4]arene derivatives ***p*-TCA-2** facing each other are symmetrical with respect to a plane and thus form two groups. The ligands of the first group (Blue) are not at the same height and are turned at 90° compared to the second group (Yellow).

The side view in Figure 4 highlights the topology of the polymer. Indeed, the 1,3-alternate conformation of the ***p*-TCA-2** ligand, and the arrangement in pairs of the thiacalixarenes derivatives which is also given in Figure 6, give a wavy structure to the two-dimensional coordination polymer.

Probably as a result of better packing, the 2-D wavy sheets are stacked in a slightly shifted parallel fashion, generating cavities (Figure 5). Indeed, the first sheet is shifted horizontally from the second, by a unit which represents approximately half a calix. Additionally, the interstices or cavities between these two polymers are filled with SbF_6_^−^ anions. For that reason, we observe no interaction between the different layers since the minimum distance between them is 4.3 Å.

In conclusion, using the thiacalix[4]arene derivative ***p*-TCA-2**, in the neutral form and in 1,3-alternate conformation, bearing four thiomethyl pendant groups and Ag(I) cations, a 2-D coordination network has been obtained via self-assembly process and structurally characterised in the crystalline phase based on coordination bond type interactions. Thus, we have demonstrated that thiacalixarenes could quite play the role of a rigid skeleton, on which it is quite easy to graft various coordinating units. The effect of the flexible nature of the coordinating arm on the dimensionality was demonstrated: when flexible alkane chains are used, networks with higher dimensions can be obtained.

## Electronic supporting information

### NMR characterizations

Figure S1. ^1^H NMR of *p*-TCA-1 in CDCl_3._

Figure S2. ^13^C NMR of *p*-TCA-1 in CDCl_3._

Figure S3. ^1^H NMR of *p*-TCA-2 in CDCl_3._

Figure S4. ^13^C NMR of *p*-TCA-2 in CDCl_3._

## Figures and Tables

**Figure 3:**
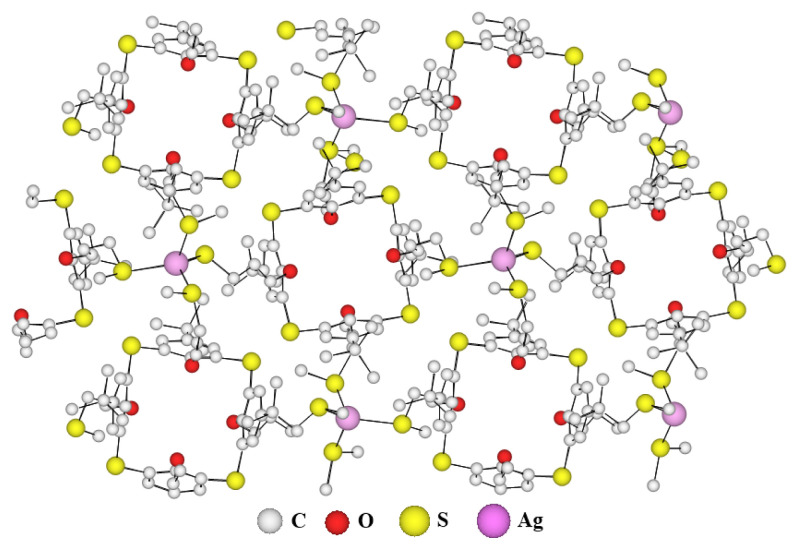
X-ray crystal structure of the 2-D network obtained with ligand ***p*-TCA-2** and AgSbF_6_. (SbF_6_^−^ anions omitted for clarity).

**Table:** Crystallographic datas for ***p*-TCA-2** and the network obtained from ***p*-TCA-2** and AgSbF6.

	*p*-TCA-2	Network
Formula	C_56_H_80_O_4_S_8_	C_61_H_92_AgF_6_O_7_S_8_Sb :C_56_H_80_Ag.SbF_6_•C_4_H_8_O_2_•CH_3_OH
Molecular weight	1073.77	1537.53
Crystal system	tetragonal	monoclinic
Space group	I 41/a	P 1 21/n 1
a(Å)	19.3952(8)	14.9907(2)
b(Å)	19.3952(8)	13.8259(2)
c(Å)	15.6769(6)	34.0796(5)
β(deg)		102.489(5)
V(Å3)	5897.2(4)	6896.2(2)
Z	4	4
Colour	colourless	colourless
Crystal dim(mm)	0.20*0.12*0.10	0.20*0.08*0.04
Dcalc(gcm^−3^)	1.21	1.48
F000	2304	3176
m(mm^−1^)	0.345	0.982
Trans. min and max	0.932/0.966	0.820/0.961
Temperature(K)	173	173
Wavelength(Å)	0.71073	0.71073
Radiation	MoKa graphite monochromated	MoKα graphite monochromated
Diffractometer	KappaCCD	KappaCCD
Scan mode	‘phi scans’	‘phi scans’
hkl limits	−25.25/−17.17/−20.15	0.20/0.18/−46.45
Theta limits(deg)	2.5/27.48	2.5/29.10
Number of data meas.	5436	19109
Number of data with I > 3 s(I)	1363	7634
Number of variables	154	703
R	0.107	0.111
Rw	0.117	0.122
GOF	1.017	1.006
Largest peak in final difference (eÅ^−3^)	0.489	1.053
