# Supplementary figures and images for: New 2-D silver(I) coordination network constructed from thiomethyl group-substituted p-tert-butylthiacalix[]arene
Source: Turk J Chem. 2022 May 11;46(5):1541–7. doi: 10.55730/1300-0527.3459 (PMC10390205; doi:10.55730/1300-0527.3459)

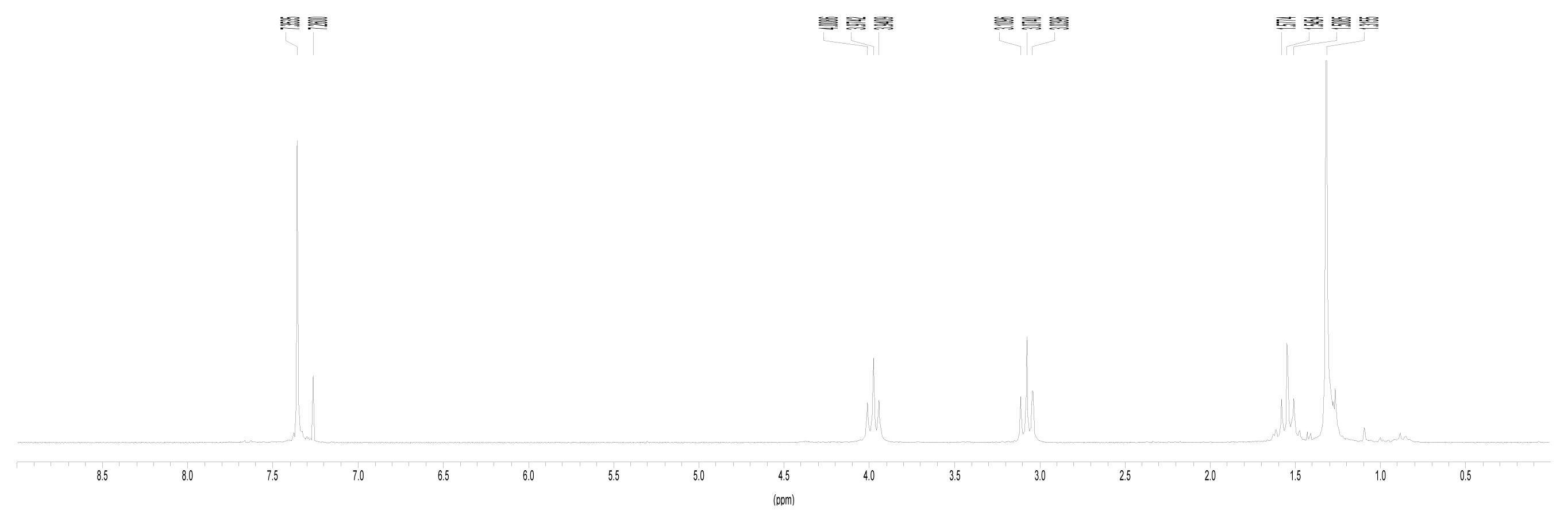

Supplement: Figure S1. — 1H NMR of p-TCA-1 in CDCl3. [file turkjchem-46-5-1541s1.tif]

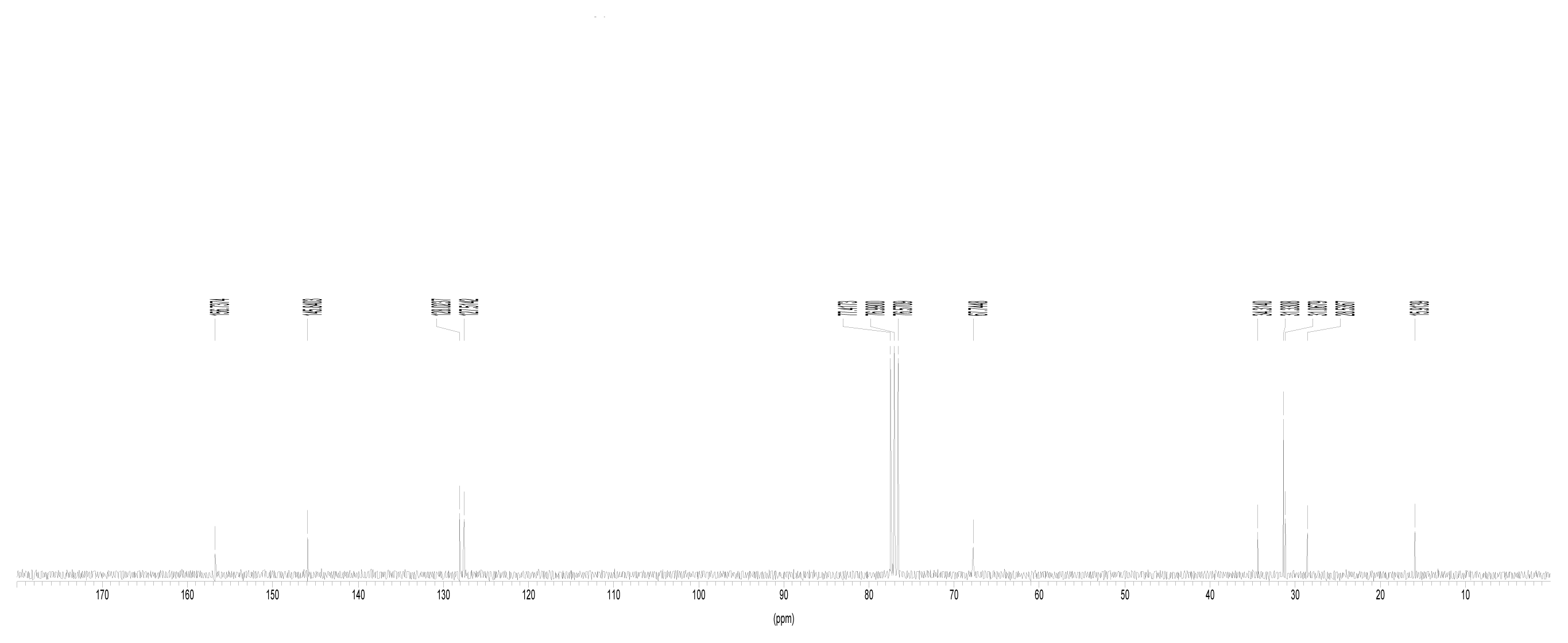

Supplement: Figure S2. — 13C NMR of p-TCA-1 in CDCl3. [file turkjchem-46-5-1541s2.tif]

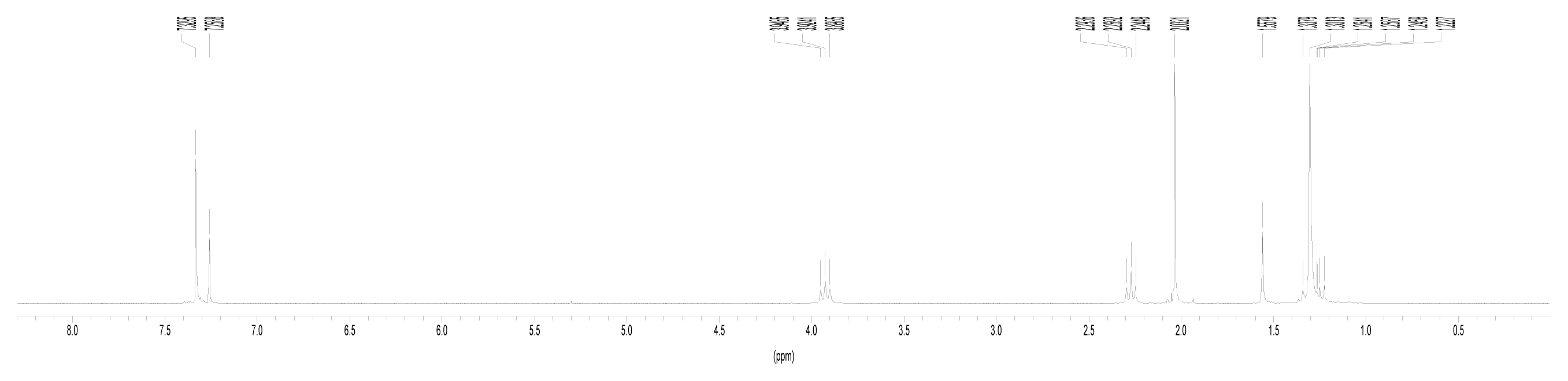

Supplement: Figure S3. — 1H NMR of p-TCA-2 in CDCl3. [file turkjchem-46-5-1541s3.tif]

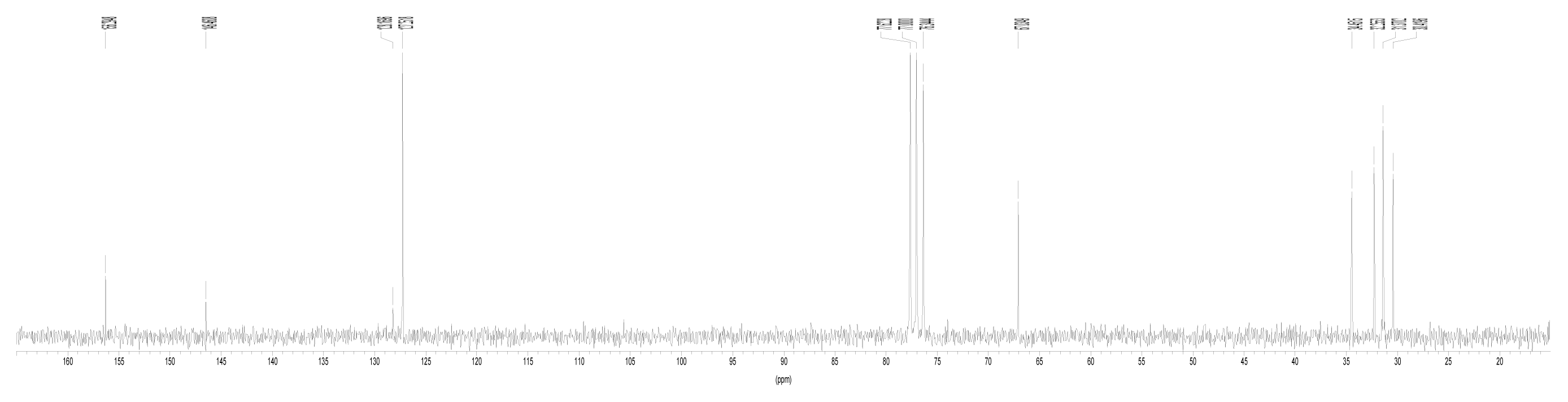

Supplement: Figure S4. — 13C NMR of p-TCA-2 in CDCl3. [file turkjchem-46-5-1541s4.tif]
